# The role of glymphatic system in brain injury after subarachnoid hemorrhage

**DOI:** 10.3389/fimmu.2026.1953626

**Published:** 2026-09-17

**Authors:** Junli Peng, Wanying Li, Qian Zhong, Liwei Li, Sufang Tang, Yiheng Li, Qiang Liu

**Affiliations:** 1 Department of Brain Diseases, Shuangfeng County Hospital of Traditional Chinese Medicine, Loudi, Hunan, China; 2 Xiangya School of Medicine, Central South University, Changsha, Hunan, China; 3 School of Health Medicine, Kashgar Vocational and Technical College, Xinjiang, China; 4 School of Pharmacy, College of Medicine, Hunan Normal University, Changsha, Hunan, China; 5 Guangzhou Institute of Respiratory Health, First Affiliated Hospital of Guangzhou Medical University, Guangzhou, China; 6 Department of Neurology, First Affiliated Hospital of Guangzhou Medical University, Guangzhou, China; 7 Department of Neurology, Loudi Hospital of the First Affiliated Hospital of Guangzhou Medical University, Loudi, Hunan, China

**Keywords:** aquaporin-4 (AQP4), delayed cerebral ischemia (DCI), early brain injury (EBI), glymphatic system, subarachnoid hemorrhage

## Abstract

Subarachnoid hemorrhage (SAH) remains a devastating cerebrovascular emergency with high mortality and long-term neurological morbidity, yet the mechanisms driving secondary brain injury remain incompletely understood. The glymphatic system, a glial-dependent perivascular clearance pathway, promotes convective exchange of cerebrospinal fluid and interstitial fluid through aquaporin-4 water channels on astrocytic endfeet to remove neurotoxic solutes. Emerging evidence implicates glymphatic dysfunction as a key mediator of brain injury after SAH. This review synthesizes current knowledge on the anatomical and physiological foundations of the glymphatic system, examines the multifaceted mechanisms by which SAH disrupts this clearance network, and discusses the pathophysiological consequences linking glymphatic impairment to neurological outcomes. Understanding the glymphatic dimension of SAH pathophysiology may illuminate novel therapeutic targets and ultimately improve the clinical management of this devastating condition.

## 1 Introduction

Subarachnoid hemorrhage (SAH) is a devastating cerebrovascular emergency with high mortality and most survivors left with significant long-term disability (1, 2). Early brain injury (EBI) occurs within the first 72 h, delayed cerebral ischemia (DCI) commonly occurs 3–14 days post-SAH (1, 3). The secondary brain injury after SAH arises from a complex interplay of multiple mechanisms, including microcirculatory dysfunction (4, 5), cortical spreading depolarizations (6–8), neuroinflammation (9–11), oxidative stress (12, 13), blood-brain barrier disruption (14, 15), and impaired cerebral waste clearance (16, 17). Among these emerging mechanisms, the glymphatic system—a perivascular clearance pathway that removes neurotoxic solutes from brain—has garnered increasing attention as a potential unifying link between the acute deposition of blood products in the subarachnoid space and the delayed development of neurological sequelae (18, 19).

The glymphatic system facilitates the bulk flow exchange of cerebrospinal fluid (CSF) and interstitial fluid (ISF) through the brain parenchyma, enabling the efficient removal of neurotoxic solutes, including amyloid-β, tau, and various metabolic byproducts (20–23). Beyond its homeostatic role, the glymphatic system is increasingly recognized as a critical determinant of brain health, with its impairment implicated in the pathogenesis of Alzheimer’s disease (22, 24, 25), Parkinson’s disease (26, 27), traumatic brain injury (28, 29), and acute cerebrovascular events (30, 31), positioning it as a promising therapeutic target and a unifying concept for understanding the intersection of vascular health, neuroinflammation, and neurodegeneration.

## 2 Anatomical and physiological foundations of the glymphatic system

### 2.1 Structural organization of the glymphatic pathway

The glymphatic pathway comprises three interconnected anatomical compartments that enable directional CSF-ISF exchange and waste clearance (20). The para-arterial influx route allows CSF from the subarachnoid space to enter the brain parenchyma along perivascular spaces surrounding penetrating arteries, driven primarily by arterial pulsatility (32, 33). The trans-parenchymal pathway constitutes the core exchange mechanism, wherein incoming CSF mixes with ISF through the brain interstitium, a process critically dependent on aquaporin-4 (AQP4) water channels highly polarized on astrocytic endfeet that ensheath the cerebral vasculature (34, 35). The para-venous efflux route then drains the CSF-ISF mixture, now containing cleared metabolic wastes, along perivenous spaces to meningeal lymphatic vessels and ultimately to deep cervical lymph nodes (36). This hierarchical organization ensures directional bulk flow distinct from passive diffusion, and disruption at any of these three compartments can severely compromise waste clearance and predispose the brain to injury.

### 2.2 Physiological regulation of glymphatic function

Glymphatic function is dynamically regulated by multiple physiological factors that together determine the efficiency of CSF-ISF exchange and waste clearance. Arterial pulsatility—the rhythmic expansion and contraction of vessel walls driven by the cardiac cycle—serves as the primary driving force for para-arterial CSF influx (32), and any reduction in pulsatility, such as that occurring with aging, hypertension or vasospasm, significantly impairs glymphatic flow (33, 34). The sleep-wake cycle exerts a profound modulatory effect: glymphatic activity is substantially enhanced during sleep, associated with a 60% expansion of the extracellular space that reduces tortuosity for solute movement, coupled with reduced noradrenergic tone that permits greater interstitial fluid volume (37). Additionally, norepinephrine released from locus coeruleus drives slow vascular movement and plays a crucial role in brain clearance during sleep (38). Body posture also influences glymphatic clearance, with lateral positioning appearing to facilitate more efficient drainage compared to prone or supine positions, likely due to gravitational effects on CSF dynamics (39). Neuroinflammation exerts a complex effect to glymphatic clearance: acute inflammatory stimuli significantly reduced glymphatic flow without AQP4 alteration (40), whereas sustained or chronic inflammation typically impaired glymphatic activity via AQP4 depolarization (41). This multifactorial regulation underscores the vulnerability of the glymphatic system to pathological states that disrupt any of these physiological determinants, and highlights potential therapeutic avenues—such as sleep optimization and blood pressure management—for preserving glymphatic function in brain injury.

## 3 Mechanisms of glymphatic dysfunction after SAH

Following the initial hemorrhage, the subarachnoid space is inundated with a complex mixture of blood products, coagulation factors, and cellular debris that collectively pose a multifaceted threat to the structural and functional integrity of the glymphatic pathway. The mechanisms by which SAH disrupts glymphatic clearance are not unitary but rather converge through at least five interrelated routes (**Figure 1**).

**Figure 1:**
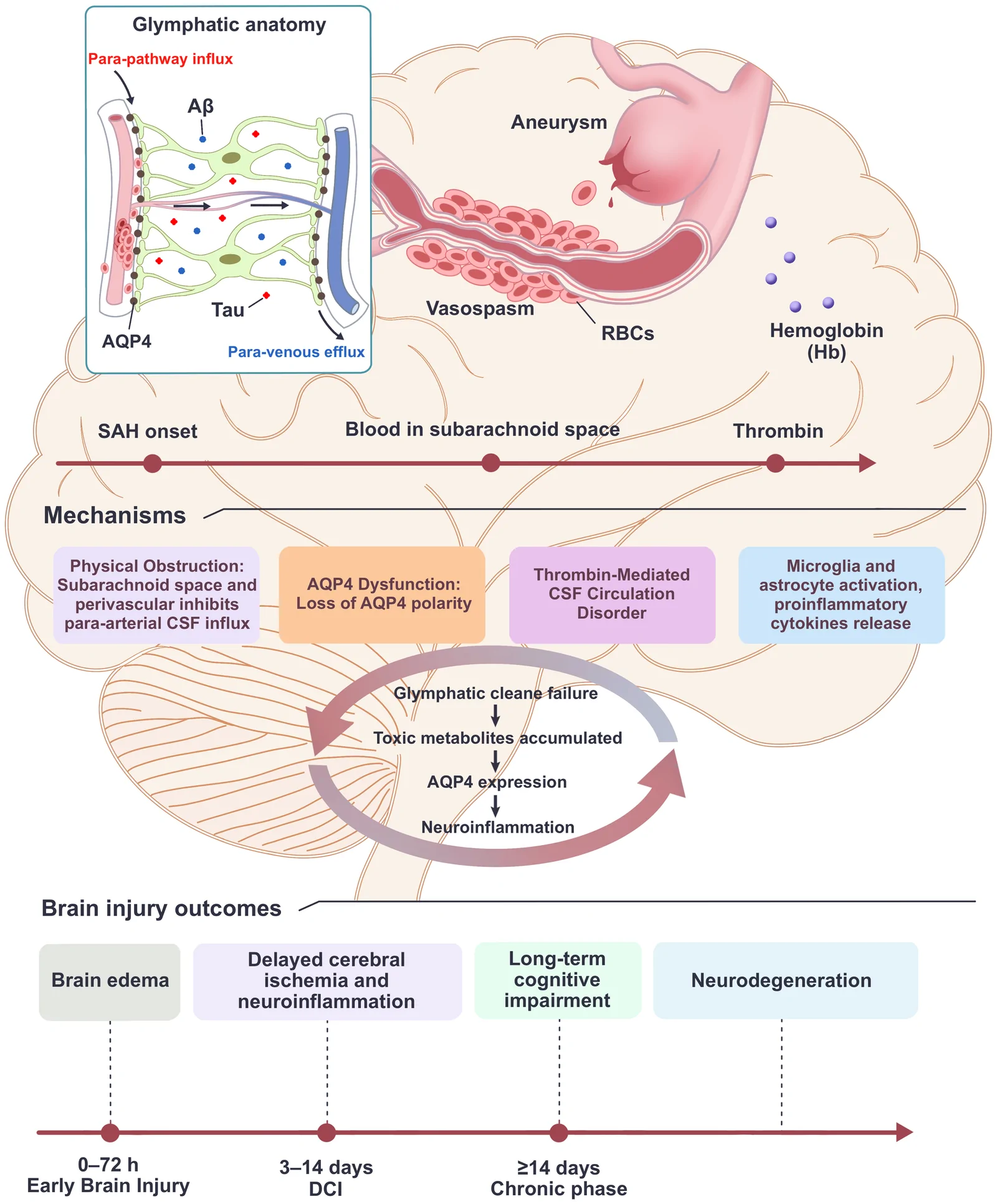
Schematic overview of glymphatic dysfunction and its pathological consequences following subarachnoid hemorrhage (SAH). The diagram illustrates the temporal cascade from SAH onset to brain injury outcomes. Following aneurysm rupture, blood products (red blood cells, hemoglobin) enter the subarachnoid space, triggering multiple mechanistic pathways: physical obstruction of perivascular influx, loss of aquaporin-4 (AQP4) polarity, thrombin-mediated cerebrospinal fluid circulation disorder, and activation of microglia and astrocytes with release of proinflammatory cytokines. These events collectively lead to glymphatic clearance failure, accumulation of toxic metabolites (including Aβ and tau), and sustained neuroinflammation, which in turn exacerbate AQP4 dysfunction in a putative vicious cycle. Clinically, this cascade contributes to early brain injury (0–72 h), delayed cerebral ischemia (DCI, 3–14 days), and long-term cognitive impairment (≥14 days, chronic phase). Red arrows indicate pathogenic progression; dashed lines represent hypothesized feedback mechanisms. AQP4, aquaporin-4; CSF, cerebrospinal fluid; RBC, red blood cell.

### 3.1 Direct physical obstruction by blood products

SAH introduces a massive burden of erythrocytes, hemoglobin, fibrinogen, and other blood components into the CSF space (42, 43). These blood products directly obstruct the perivascular spaces that constitute the glymphatic influx and efflux routes (18). In an established mouse model of SAH via blood injection into the prechiasmatic cistern, erythrocytes was observed to infiltrate perivascular spaces and brain tissues (44), with concurrent expansion of perivascular spaces indicating structural compromise of the glymphatic pathway. Similarly, a rat SAH model using endovascular perforation demonstrated marked expansion of perivascular spaces and significantly decreased ISF clearance rates, accompanied by apoptosis of endothelial cells, activation of astrocytes, overexpression of matrix metalloproteinase 9, and loss of collagen type IV—all hallmarks of intramural periarterial drainage (IPAD) failure (45). Importantly, using dynamic contrast-enhanced MRI in a beagle SAH model, researchers observed that perivascular spaces progressively enlarged over time, indicating that SAH causes irreversible structural damage to the glymphatic system, even as functional impairment may partially recover (19).

### 3.2 AQP4 dysfunction: the molecular hub

AQP4 water channels, densely polarized on astrocytic endfeet ensheathing the cerebral vasculature, are essential for glymphatic CSF-ISF exchange (46, 47). AQP4 polarity loss as functional marker of glymphatic clearance impairment (35). In a mouse SAH model (autologous blood injection into cisterna magna), the polarization of astrocytic AQP4 was impaired at one week post-hemorrhage (48). This loss of polarized expression reduces the directional water flux necessary for convective solute clearance. A recent study established the triggering receptor expressed on myeloid cells-1 (TREM1) upregulates astrocytic AQP4 expression, and the “TREM1–AQP4” axis as a critical bridge linking neuroinflammation and cerebral edema after SAH (49). Furthermore, the damage of glymphatic system induced by SAH were exacerbated in Aqp4 gene knockout (Aqp4^−/−^) rat (50). Importantly, loss of perivascular AQP4 polarity is a necessary but not sufficient indicator of impaired glymphatic transport, and that it can equally reflect astrocytic swelling or edema formation independent of clearance function.

### 3.3 Neuroinflammation as a driver of glymphatic failure

Neuroinflammation is both a cause and a consequence of glymphatic dysfunction after SAH. The subarachnoid deposition of blood degradation products—particularly hemoglobin—triggers a robust innate immune response (51). In the acute phase (within 24 hours), skull-derived neutrophils infiltrate the brain and primed for NETosis (52). After SAH, activated glial cells release pro-inflammatory cytokine IL-6, which further impair glymphatic function through multiple mechanisms: direct damage to perivascular architecture, disruption of AQP4 polarization, and promotion of endothelial dysfunction (49). The relationship is bidirectional and self-perpetuating: glymphatic dysfunction leads to accumulation of neurotoxic substances (including hemoglobin breakdown products, inflammatory mediators, and tau proteins), which sustain and amplify neuroinflammation; sustained neuroinflammation further impairs glymphatic clearance by disrupting AQP4 function and perivascular integrity (53).

### 3.4 Thrombin-mediated CSF circulation disorder

The coagulation cascade is activated immediately upon blood entering the subarachnoid space, generating thrombin (54, 55). While the precise molecular mechanisms require further elucidation, thrombin may damage the glymphatic-meningeal lymphatic system through multiple pathways: direct proteolytic effects on perivascular basement membranes, induction of endothelial dysfunction, and promotion of fibrin deposition that physically obstructs CSF outflow pathways (54).

### 3.5 Vasospasm reduces arterial pulsatility

Pial microvasospasm, manifesting as sustained constriction of the cerebral vasculature, constitutes a critical pathogenic event that reductions of cerebral blood flow (CBF) and precipitates cerebral ischemia in the post-SAH period (18, 56, 57). Identifying the mechanisms of pial vasospasm may therefore result in novel therapeutic options for SAH patients. It was found that the perivascular macrophages (PVM) contribute to the formation of microvasospasms after experimental SAH, depletion of PVM via clodronate-loaded liposomes could significantly reduced the number of microvasospasms (58). Following SAH, neutrophils infiltrate the perivascular space and form neutrophil extracellular traps (NETs), which are associated with the development of pearl-string-like microvasospasms in the adjacent pial arterioles. Conversely, neutrophil depletion significantly reduces the incidence of these microvasospasms (59).

Arterial pulsatility is the primary driving force for para-arterial CSF influx (32). Then large-vessel vasospasm and microvascular spasm, by attenuating the arterial pulsatility, result in a significant reduction in cortical perfusion after SAH (57, 60). Moreover, microvascular spasm in the hippocampus may further impair local glymphatic function in regions critical for cognitive function.

## 4 Glymphatic dysfunction and SAH pathophysiology

### 4.1 Early brain injury

Early brain injury (EBI) encompasses the acute pathophysiological events occurring within the first 72 hours after SAH and directly impacts patient mortality (61). Glymphatic impairment is predominantly a consequence of SAH-induced rises in intracranial pressure, loss of arterial pulsation, and acute AQP4 depolarization. Using an AQP4 knockout rat model established via the endovascular perforation method, researchers demonstrated that AQP4 deficiency significantly increased whole-brain water content and aggravated neurological deficits following SAH (50).

The deleterious role of glymphatic dysfunction in EBI is further underscored by studies on haptoglobin (Hp), a hemoglobin scavenger protein. In a mouse SAH model established by blood injection into the prechiasmatic cistern, Hp administration decreased mortality and brain edema until 48 hours post-SAH, suppressed neurological impairments, reduced hemoglobin infiltrations into perivascular spaces and brain parenchyma, attenuated fibrinogen-positive microthrombi formation, and inhibited microglial activation and caspase-dependent neuronal apoptosis (62). Critically, immunohistochemical staining of deep cervical and mandibular lymph nodes demonstrated that hemoglobin was increased in the lymphatic sinus after SAH and was further increased by Hp administration in SAH animals, indicating that Hp protects against EBI by increasing lymphatic drainage of free hemoglobin (62). This provides direct evidence that enhancing glymphatic-meningeal lymphatic efflux capacity can limit the neurotoxic effects of blood products during the EBI window.

### 4.2 Delayed cerebral ischemia

Delayed cerebral ischemia, occurring between days 4 and approximately 14 after SAH onset, is a major predictor of poor outcomes and is currently understood as a multifactorial process that evolves over time (63, 64). Modern advances have established that DCI arises from a complex interplay of vascular dysfunction, inflammation, glymphatic impairment, and neuroelectric disruption (64). Glymphatic failure becomes an amplifier of injury by reducing clearance of pro-inflammatory metabolites (e.g., hemoglobin, fibrinogen, DAMPs), thereby worsening edema and neuroinflammation. Murine studies demonstrate that glymphatic and meningeal lymphatic dysfunction persists for at least one week post-SAH, associated with Tau protein accumulation, lymphocyte infiltration (CD3^+^, CD4^+^, and CD8^+^ cells), microvascular spasm, glial cell activation, neuroinflammation, and neuronal apoptosis in the hippocampus (48). This persistent dysfunction overlaps with the peak period for DCI development, suggesting that glymphatic impairment may both predispose to and perpetuate delayed ischemic injury.

### 4.3 Cerebral edema

Cerebral edema is a major complication of SAH and an independent predictor of poor prognosis (65). Glymphatic dysfunction critically contributes to edema formation through multiple mechanisms. Loss of AQP4 polarization impairs CSF-ISF exchange and water homeostasis, leading to accumulation of excess fluid in the brain parenchyma (66). Blood-brain barrier disruption, which is exacerbated by glymphatic dysfunction, allows plasma leakage into the brain interstitium, contributing to vasogenic edema (30). The therapeutic implications of glymphatic modulation for edema reduction are supported by recent experimental evidence. In a rabbit model of SAH using a modified two-hemorrhage cisterna magna injection, both nimodipine and enoxaparin were associated with improved neurological outcomes and reduced brain edema (67). Both treatments may modulate perivascular clearance through mechanisms independent of AQP4 transcriptional regulation.

### 4.4 Perpetuation of neuroinflammation by glymphatic failure

The relationship between glymphatic dysfunction and neuroinflammation in SAH is bidirectional and self-perpetuating. Blood components rapidly enter the paravascular space and penetrate into the parenchyma, causing widespread perivascular neuroinflammation (18). Activated glial cells release pro-inflammatory cytokines that further impair glymphatic function through direct damage to perivascular architecture and disruption of AQP4 polarization. Thrombin, generated during the coagulation cascade activated by subarachnoid blood, contributes to CSF circulation disruption by damaging the glymphatic-meningeal lymphatic system, leading to cerebral edema, hydrocephalus, cognitive impairments, and depressive symptoms (54).

### 4.5 Hydrocephalus

Post-hemorrhagic hydrocephalus is a common complication of SAH that significantly impacts patient outcomes. Glymphatic dysfunction contributes to hydrocephalus through impaired CSF circulation and clearance (68, 69). The accumulation of blood products and thrombin in the subarachnoid space obstructs traditional CSF outflow pathways while simultaneously damaging the glymphatic and meningeal lymphatic drainage pathways.

### 4.6 Cognitive impairment

Persistent cognitive impairment is a major cause of disability in SAH survivors, affecting up to 50% of patients (70). Glymphatic dysfunction likely contributes to cognitive decline through accumulation of neurotoxic proteins, sustained neuroinflammation, and white matter injury (21, 71). One week post SAH, persistent glymphatic dysfunction was associated with accumulation of tau proteins in the mouse brain (48). Higher total tau levels in the cerebral microdialysate may be a useful biomarker associated with cognitive decline in poor-grade subarachnoid haemorrhage patients (72). Dramatic reduction in the length density of AQP4 positive capillaries early after SAH may contribute to the atrophy in hippocampus, a region critical for memory and cognitive function (73).

## 5 Glymphatic impairment in SAH patients: clinical evidence from contrast-enhanced MRI imaging and DTI-ALPS studies

It was found that the MRI imaging contrast-gadobutrol or gadolinium could distribute throughout the brain after intrathecal administration, become a useful tool to study glymphatic function in the human brain (74–77). With gadolinium-based MRI imaging, the patients showed the marked glymphatic impairment from 24 hours post-SAH, and were most pronounced after 3 to 6 months (78).

The DTI-ALPS index (without injection of contrast agent) is a non-invasive MRI metric derived from diffusion tensor imaging that evaluates directional water diffusivity in the deep white matter to infer perivascular CSF dynamics (79–81). A reduced DTI-ALPS index—indicating decreased water diffusivity along perivascular spaces—is interpreted as reflecting impaired glymphatic function. Importantly, the DTI-ALPS index has been validated against gold-standard gadolinium-based glymphatic imaging, demonstrating strong correlations that support its use as a surrogate marker (82).

A landmark retrospective study of 60 patients with aneurysmal SAH provided the systematic evidence that glymphatic dysfunction occurs in human patients and has prognostic significance (83). Patients with poor functional outcomes at 3 months (modified Rankin Scale score ≥3) had significantly lower DTI-ALPS index values compared with those who achieved favorable outcomes. Importantly, patients who developed shunt-dependent hydrocephalus, exhibited a markedly reduced DTI-ALPS index (83). A subsequent multimodal MRI study in 58 SAH patients and 41 controls revealed not only a reduced DTI-ALPS index, but also increased free water in white matter and an elevated perivascular space volume fraction—all of which are consistent with glymphatic dysfunction and interstitial fluid retention (84).

DTI-ALPS index with some technical limitations: sensitivity to bulk motion, partial volume effects, dependence on specific fiber orientation, and lack of standardized acquisition protocols across centers. DTI-ALPS indexes water diffusivity along perivascular spaces but does not directly measure convective flow or clearance of specific solutes. It can be confounded by vasogenic edema, white matter integrity changes, and even age-related atrophy.

## 6 Therapeutic implications and emerging strategies

The mechanistic framework linking glymphatic dysfunction to SAH pathophysiology has opened multiple therapeutic avenues. These strategies can be conceptually organized into two tiers (1): strategies targeting the AQP4 modulation (2); strategies improving cerebral blood flow or arterial pulsatility. It should be noted that, pharmacological studies (BHB, PACAP, et al) have only been validated in animal SAH models, not the clinical research.

### 6.1 Preserving polarity and expression of AQP4 to maintain CSF-ISF exchange

β-hydroxybutyrate (BHB), a ketone body endogenously produced during fasting or ketogenic diets, has recently emerged as a promising agent for restoring glymphatic function (85). In a murine SAH model established by cisterna magna blood injection, BHB treatment reinstated AQP4 polarization by upregulating SNTA1 (syntrophin-alpha-1) protein—a critical anchoring protein that maintains AQP4’s perivascular localization (86). This restoration of AQP4 polarity enhanced glymphatic system function, as demonstrated by improved tracer clearance.

### 6.2 Improving cerebral blood flow or arterial pulsatility

#### 6.2.1 Nimodipine: beyond vasospasm prevention

Nimodipine, the only pharmacologic agent with proven efficacy in improving outcomes after aneurysmal SAH, has long been assumed to act primarily through prevention of cerebral vasospasm (87–89). However, accumulating evidence suggests that its neuroprotective effects may be partially mediated through glymphatic modulation. In a mouse SAH model, nimodipine administration improved glymphatic function and eliminated the effects of edema and neurological dysfunction after SAH, suggest a neuroprotective role for nimodipine that extends beyond its conventional vasospasm-preventing effects (90). A rabbit model of SAH using a modified two-hemorrhage cisterna magna injection demonstrated that nimodipine treatment was associated with improved neurological scores and food intake. Histologically, nimodipine reduced the perivascular-to-vascular area ratio, reflecting attenuated perivascular fluid accumulation—a structural correlate of glymphatic obstruction (67).

#### 6.2.2 Enoxaparin: pleiotropic effects on perivascular clearance

Enoxaparin, a low-molecular-weight heparin, demonstrated safe and effective in reducing cerebral vasospasm and ischemia after SAH (91). Like nimodipine, enoxaparin reduced the perivascular-to-vascular area ratio, suggesting modulation of glymphatic perivascular spaces (67). The absence of AQP4 alteration indicates that enoxaparin’s glymphatic effects may be mediated through mechanisms such as reduced fibrin deposition, anti-inflammatory properties, or preservation of perivascular basement membrane integrity (67).

#### 6.2.3 Tissue plasminogen activator

Tissue-type plasminogen activator (tPA) is a potential therapeutic agent for improving glymphatic clearance following SAH. In murine SAH models, intraventricular tPA administration promoted fibrinolysis of subarachnoid blood clots within the perivascular compartment, thereby restoring glymphatic clearance and leading to improved neurological outcomes by day 7 post-SAH (18). In another study with SAH mice, intracisternal injection of tPA lowered intracranial pressure, increased cortical blood volume, and partially restored CSF flow at 24 h after SAH (92). These studies suggesting that fibrinolytic agents may relieve glymphatic obstruction by clearing fibrin deposits from perivascular spaces. Prospective randomized studies suggested that intrathecal or intraventricular injection of tPA is effective and safe in reducing vascular spasm and delayed ischemic defects in patients with aneurysmal SAH (93, 94).

#### 6.2.4 Pituitary adenylate cyclase-activating polypeptide

PACAP is a pleiotropic neuropeptide that signals through PAC1, VPAC1, and VPAC2 receptors to regulate vascular tone, cerebral blood flow, and neuronal survival (95). Its neuroprotective and vasodilatory properties have attracted considerable interest in the context of acute brain injury, including SAH. Treatment with PACAP has been shown to increase glymphatic function after SAH and alleviate subsequent increases in intracranial pressure and brain water content, representing another potential therapeutic avenue (96).

## 7 A roadmap for clinical translation

A coordinated four-phase roadmap can accelerate translation of glymphatic mechanisms into SAH clinical practice. Phase 1 focuses on standardizing non-invasive tools: multi-center validation of DTI-ALPS against gold-standard intrathecal contrast MRI, establishing acquisition protocols, and developing automated analysis pipelines to enable integrated assessment of the glymphatic-meningeal lymphatic axis across institutions. Phase 2 aims to develop a multi-analyte biomarker panel—including CSF/serum inflammatory cytokines, microparticle profiles, tau and amyloid-β levels, blood-brain barrier integrity markers, and thrombin-related markers—evaluated longitudinally from hyperacute to chronic phases to construct composite prognostic models. Phase 3 seeks to define temporal therapeutic windows by delineating the trajectory of glymphatic dysfunction across EBI (0–72h), DCI (3–14d), and chronic stages, while validating risk-stratification algorithms to enable personalized intervention decisions. Phase 4 involves designing rational clinical trials that combine therapies targeting multiple pathogenic nodes simultaneously (e.g., nimodipine plus anti-inflammatory agents), employ adaptive designs, enrich patient populations using validated biomarkers, and prioritize functional and cognitive endpoints over surrogate measures. This iterative, collaborative framework—integrating neurointensivists, neuroradiologists, immunologists, and biostatisticians—provides a realistic pathway toward reducing the neurological burden of SAH through glymphatic-targeted therapeutics.

## 8 Conclusion

The glymphatic system plays a pivotal role in the pathophysiology of brain injury after SAH. SAH disrupts glymphatic function through multiple mechanisms: physical obstruction, AQP4 dysfunction, thrombin-mediated damage, neuroinflammation, and mLV impairment. Glymphatic dysfunction contributes to EBI, DCI, cerebral edema, hydrocephalus, neuroinflammation, and cognitive impairment. Current clinical evidence from imaging studies supports the translational relevance of glymphatic mechanisms in SAH patients. Therapeutic strategies targeting glymphatic system offer promising avenues for improving outcomes in SAH patients. Future research should focus on: validating clinical biomarkers; defining therapeutic windows; understanding reversibility; and developing targeted interventions.
